# An Integrated Laser-Induced Piezoelectric/Differential Confocal Surface Acoustic Wave System for Measurement of Thin Film Young's Modulus

**DOI:** 10.3390/s120912208

**Published:** 2012-09-05

**Authors:** Fei Yang, Dante J. Dorantes-Gonzalez, Kun Chen, Zimo Lu, Baoyin Jin, Yanning Li, Zhi Chen, Xiaotang Hu

**Affiliations:** State Key Laboratory of Precision Measuring Technology and Instruments, Tianjin University, Weijin Road, No.92, Tianjin 300072, China; E-Mails: ydishikun@163.com (F.Y.); chenkun789@yahoo.cn (K.C.); zimo.lu@yahoo.cn (Z.L.); bao420487704@163.com (B.J.); ch-z@163.com (Z.C.); xthu@tju.edu.cn (X.H.)

**Keywords:** Young's modulus, laser-induced surface acoustic waves, piezoelectric, differential confocal

## Abstract

The present paper presents the design and development results of a system setup for measuring Young's modulus of thin films by laser-induced surface acoustic waves based on the integration of two detection methods, namely, piezoelectric transducer detection and differential confocal detection, which may be used for conducting consecutive or simultaneous measurements. After demonstrating the capabilities of each detection approach, it is shown how, depending on a wider range of applications, sample materials and measurement environments, the developed integrated system inherits and harnesses the main characteristics of its detection channels, resulting in an more practical and flexible equipment for determining Young's modulus than traditional nanoindentation equipment, and also suitable for cross-validation purposes.

## 1. Introduction

Thin films technology is widely used in the optical, semiconductor, biomedical and many other fields. Thin films' mechanical properties, such as Young's modulus and hardness, determine the performance of products to a certain extent, which makes the measurement of Young's modulus and other mechanical properties a significant research topic [1–4]. Currently, the main techniques for measuring Young's modulus of thin films are the following: bending testing [1], nanoindentation [5], Brillouin scattering [6], and surface acoustic wave (SAW) spectroscopy [1]. The present research focuses particularly on the laser-induced surface acoustic wave (LSAW) technique, since this is a promising technique with the potential to perform non-contact, rapid and accurate measurements of Young's modulus in thin films, while it may be also suitable for *in situ* and on-line measurement in industrial production environments [7].

Surface acoustic waves are a type of elastic wave that propagates along the surface of half-space solids. SAW energy is concentrated near the surface, decaying exponentially with the increase of depth. SAW decay amplitude is influenced by frequency: the higher the frequency is, the higher the energy focused on the sample's surface. Surface waves can also propagate in layered solid media [1].

The laser-induced surface acoustic wave technique uses a pulse laser to excite broadband surface waves in the sample surface up to GHz and even a few THz ranges of frequencies [8]; then the excited surface waves are detected by a SAW detection technique, usually with a lower bandwidth depending on the quality of the sensor and acquisition subsystem, and finally the SAW propagation dispersion curves are analyzed to determine the Young's modulus of the sample under test.

The quality of the surface acoustic waves depends on the material's properties, the characteristics of the excitation laser, as well as on the detection technique. The laser-induced surface acoustic waves, which are excited under a thermoelastic effect, have the properties of having good reproducibility, and suitability for non-destructive testing of sample surfaces [9]. As for the main SAW detection techniques at present, we can mention the piezoelectric detection technique based on PVDF foil transducers, as well as different interferometer detection techniques, e.g., the laser Doppler detection technique, and the light reflection interferometer techniques, e.g., the differential confocal LSAW technique [10–12]. Usually, the optical interference and laser Doppler methods have a measurement bandwidth of about 50 MHz or less [13]. As a result, the narrow frequency bandwidth of these techniques makes their use to detect SAWs at high frequencies difficult, which significantly influences the final measurement accuracy [14]. The piezoelectric LSAW detection technique based on PVDF foil transducers is one of the most promising techniques at present [1,7,13], which has shown the characteristics of a higher measurement bandwidth up to 120 MHz [7], in some cases up to about 300 MHz [15,16], high signal-to-noise ratio, and a relative error in the range of 1% [1]. The differential confocal LSAW detection technique based on the principle of laser beam reflection has the advantages of having high sensitivity, short response time, and a measurement bandwidth extended up to 300 MHz [10], what's more, the technique is a promising detection method in non-destructive and non-contact detection testing, which is suitable for production environments, for example, for testing integrated circuits, requiring an ultra-clean testing environment. Hence, the piezoelectric, and the differential confocal LSAW detection techniques present major advantages in Young's modulus measurement of thin films.

This paper analyzes the advantages and disadvantages of these two techniques, and accordingly proposes the design and construction of an integrated system prototype, as well as provides the guidelines for its proper use with different sample materials and measurement scenarios.

## 2. Piezoelectric LSAW Detection Technique and Differential Confocal LSAW Detection Technique

### 2.1. Piezoelectric LSAW Detection Technique Based on A PVDF Foil Transducer

The piezoelectric LSAW detection technique is a classic method in laser ultrasonic detection technology. PVDF foils have a good acoustic impedance matching, wide measurement bandwidth, and high sensitivity for force-electric charge conversion, which makes this technique an excellent measurement method [17]. Moreover, piezoelectric foil transducers can be homemade easily [18]. For our experimental setup, a number of simple foil piezoelectric transducers were homemade, as well as innovative foil piezoelectric transducer structures [19].

The schematic diagram of the piezoelectric SAW detection system is relatively simple. In the system, a pulse laser is used to generate wideband surface acoustic waves. And then the surface acoustic waves are detected by a wideband piezoelectric transducer at different distances between the laser focus line focused by a cylindrical lens and the transducer.

### 2.2. Differential Confocal LSAW Detection Technique Based on the Principle of Light Reflection

The differential confocal LSAW detection technique uses laser beam reflection for measuring Young's modulus of thin films. The intensity of the reflected beam light ensures the system high sensitivity and rapid response characteristics, meanwhile, the differential signal sampling eliminates common disturbances, such as optical power fluctuations of the detector, ambient air convection, and electric noise, which in turn improves system's signal-to-noise ratio and the ability to distinguish high-frequency SAW signals [11,12].

The schematic diagram of the integrated LSAW detection system described in detail later in Section 3 includes a differential confocal LSAW detection system (Channel 2) previously developed by the research team [20]. In the system, the LSAW excitation part of the differential confocal system is the same with the piezoelectric system. However, the optical detection part uses a light probe emitted by a He-Ne laser to detect surface acoustic waves. The difference between the differential confocal LSAW detection method with the classic piezoelectric blade method is that the former relies on the focus change after the movement of the light and the latter is based on the principle of the exposed light energy change from the receiving surface.

### 2.3. Comparison Test of the Piezoelectric LSAW Detection Technique and the Differential Confocal LSAW Detection Technique

**Figure 1.**
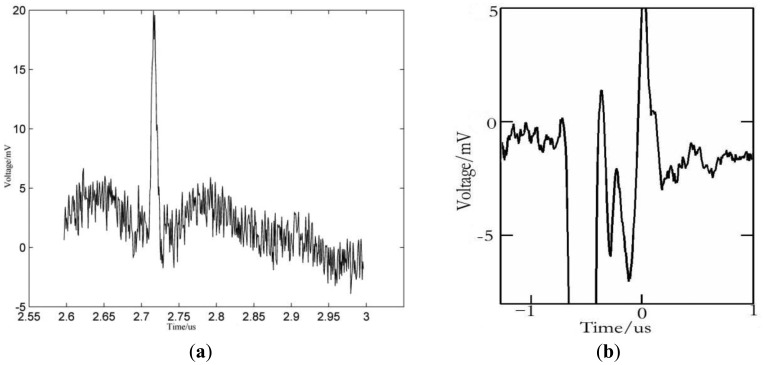
Time domain waveforms of the SAW signals obtained by (**a**) piezoelectric LSAW detection technique and (**b**) differential confocal LSAW detection technique.

In order to compare the performance of both techniques for Young's modulus measurement, a comparative experiment was conducted. A thermal oxide SiO_2_ thin film on a Si (100) substrate was chosen as experimental sample. The SiO_2_ film's density is 1,300 Kg/m^3^ measured by electron scattering, its thickness is 320 nm measured by elliptical light polarization method, and its Poisson's ratio is 0.26. The density of the Si substrate is 2,300 Kg/m^3^, its Poisson's ratio is 0.27, and its Young's modulus is 160 GPa. The time domain waveforms of the SAW signals obtained by piezoelectric LSAW and differential confocal LSAW detection techniques respectively are shown in Figure 1(a,b).

By comparing Figure 1(a,b), it can be seen that the SAW signal detected by piezoelectric LSAW detection technique has a larger amplitude, richer frequency components in general, but also has relatively significant noise amplitudes. On the other hand, the SAW signal obtained by the differential confocal LSAW detection technique shows weaker SAW signals, less frequency components, but lower noise relative amplitudes.

**Figure 2.**
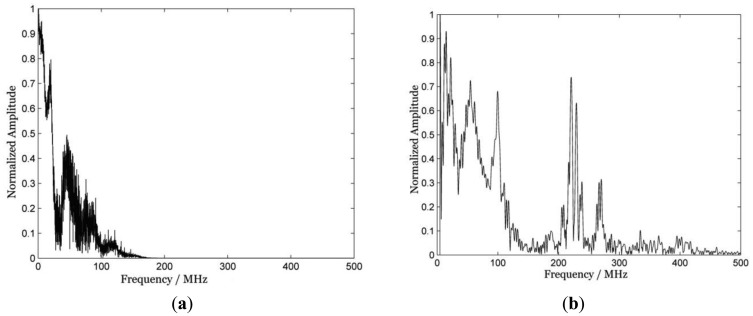
Normalized amplitude spectra of SAW signals of the piezoelectric LSAW and differential confocal LSAW detection techniques, respectively.

Figure 2(a,b) obtained as the Fast Fourier Transform of the data in Figure 1, shows the normalized amplitude spectra of the signals in Figure 1, from where the measurement bandwidth of the SAW signals can be visualized directly.

**Figure 3.**
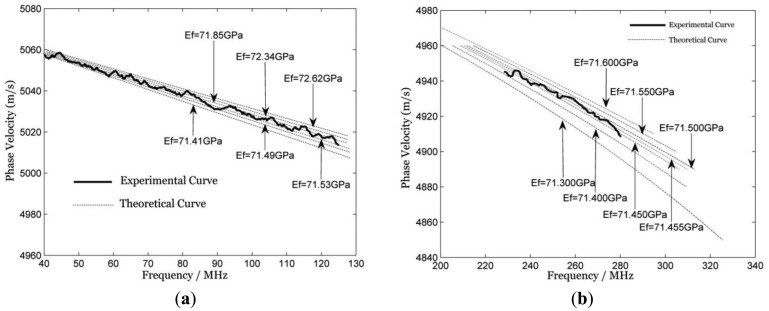
Dispersion curve fitting results of (**a**) piezoelectric LSAW detection technique and (**b**) differential confocal LSAW detection technique.

Comparing the normalized amplitude spectra, it can be seen that Figure 2(a) has a more dense composition of frequency components, but Figure 2(b) has a wider bandwidth, and the main measurement bandwidth of the piezoelectric LSAW detection technique is in the range of about 120 MHz, which is the largest bandwidth this paper can get for the limitation of the fabrication quality of the PVDF foil transducer and the oscilloscope, as well as the amplifier bandwidth, so the curve fitting between the theoretical dispersion curve and the experimental dispersion curve is to be done in the range of 40–120 MHz. Yet the differential confocal LSAW detection technique has a major bandwidth up to 300 MHz, moreover, high quality SAW signals exist in the range of 220–270 MHz, so the curve fitting is chosen to be calculated in a larger range, and consequently, it gives a larger range to fit the curve with less error. This paper used a nonlinear least-squares optimization method [3] to fit the experimental curve with the theoretical curves. The fitting results of both techniques are shown in Figure 3(a,b). The theoretical dispersion curves in the figure are derived from the equation of elastic wave motion and the boundary conditions for traction forces and displacement.

It can be seen from Figure 3(a,b) that the higher the frequency, the wider the distance among the dispersion curves. Therefore, the fitting procedure of the dispersion curves at higher frequencies leads to a higher resolution; or in other words, the higher the measurement bandwidth, the higher the resolution. On the other hand, we should take into account the large uncertainty of the Poisson ratio [2], which in our calculations was assumed fixed, since the theoretical model we used can change only one of the parameters at a time. Thus, a three digits representation will be reasonable for the representation of the Young's modulus. The Young's modulus final value of the sample measured by LSAW piezoelectric technique is 71.7 GPa with an uncertainty of 0.4 GPa, meanwhile the value measured by LSAW differential confocal technique is 71.5 GPa with an uncertainty of 0.1 GPa, hence, the differential confocal approach has a four-fold improvement in comparison with the piezoelectric detection one.

Readers should note here that the measurement improvement for Young's modulus in the case of thermal oxide SiO_2_ thin film on a Si (100) substrate is not so relevant, but when measuring the value of Young's modulus for softer materials, like soft polymers and biological interfaces, this improvement will be very helpful. It turns out that a further improvement in the accuracy of Young's modulus measurement can be obtained by improving the accuracy of Poisson ratio.

### 2.4. Advantages and Disadvantages of the Piezoelectric LSAW Detection Technique and the Differential Confocal LSAW Detection Technique

According to the experimental operation and test experiences of the authors and other research works on different samples and measurement environments, a useful systematization of the advantages and disadvantages of both techniques, the piezoelectric LSAW detection and the differential confocal LSAW detection, can be summarized in Table 1.

It can be seen from Table 1 that the piezoelectric LSAW detection technique and the differential confocal LSAW detection technique have their own intrinsic features. At present, there is a commercial piezoelectric LSAW equipment developed by a research institution in Germany [21]. This equipment uses the piezoelectric detection technology, and due to its uniqueness in the market, the cost is still similar to that of the traditional nanoindentation equipment. Then, in order to overcome the drawbacks of the piezoelectric LSAW technique, while improving the accuracy and reliability of thin film Young's modulus measurement, as well as expanding the testing performance to other measurement scenarios and samples, the authors proposed the idea of integrating the piezoelectric and the differential confocal LSAW detection techniques, aiming to take advantage of the two techniques, creating an improved procedure to measure Young's modulus of thin films. This is because, many times, the advantages of one technique are, at the same time, the disadvantages of the other, and vice versa; for example, a strong measurement signal but large noise in the piezoelectric version versus a less sensitivity but wider bandwidth in the differential confocal version.

## 3. The Integrated Laser-Induced PZT/DC SAW System for Young's Modulus Measurement of Thin Films

Both techniques, the piezoelectric LSAW detection based system, and the differential confocal LSAW detection based system, use the same excitation laser beam path to generate surface acoustic waves on the sample surface; so the new integrated system inherits this common generation subsystem. On the other hand, the structure of the piezoelectric detector is simple, and it can be integrated with the differential confocal detection subsystem to form the laser-induced piezoelectric and differential confocal LSAW system (laser-induced PZT/DC SAW system). The following section describes the components and working principle of the integrated system, as well as the testing scenarios and the operating strategies.

### 3.1. Description and Working Principle of the Integrated System Setup

**Figure 4.**
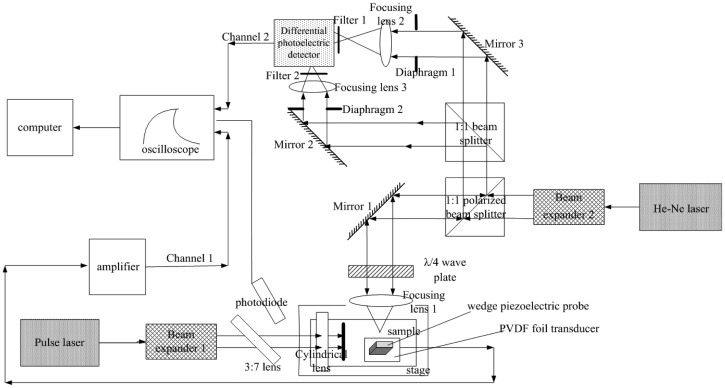
Laser beam path of the integrated laser-induced PZT/DC SAW system.

Figure 4 shows the setup schematic representation of the integrated laser-induced PZT/DC SAW system for Young's modulus measurement of thin films. The excitation light beam is emitted by a 532 nm wavelength and 800 ps pulse laser 1. After collimation and expansion 2, the pulsed beam is divided into two parts by a 3:7 beam splitter 3, of which 7/10 passes through the cylindrical lens focused on the sample surface to stimulate surface acoustic waves, and the rest is used as a trigger signal captured by the photodiode 27 to trigger the oscilloscope to make the SAW signal measurements synchronically.

As mentioned before, the surface wave detection in the integrated system consists of two parts. First, the description of the piezoelectric LSAW detection channel (Channel 1) is as follows: after the PVDF foil transducer under the wedge piezoelectric probe detects the surface acoustic wave signals. The signals are converted into electric signals, which are filtered and amplified by the amplifier, and then acquired and displayed by the oscilloscope, and finally transferred to the computer for signal processing.

The other part is related to the differential confocal LSAW detection channel (Channel 2), whose description is as follows: a probe beam, emitted by a 632.8 nm wavelength He-Ne laser, is expanded and collimated by beam expander 2, and then divided and polarized by a 1:1 polarizing beam splitter. The transmitted beam reaches the sample's surface after passing through mirror 1, λ/4 wave plate, and focusing lens 1, obtaining the SAW signals reflection information and then returning through the same path. Due to the beam passes through the λ/4 wave plate twice, its polarizing direction is changed 90°, so when it reaches the polarizing beam splitter again, it cannot be transmitted, but only reflected to a new path in the direction of the differential photoelectric detector. The reflected light comes to the 1:1 beam splitter, where it is divided into two signals. One of them goes to one input channel of the differential photoelectric detector after passing through the mirror 3, diaphragm 1, focusing lens 2 and filter 1. The other signal goes to the second input channel of the differential photoelectric detector, passing through the mirror 2, diaphragm 2, focusing lens 3, and filter 2. The two diaphragms are able to adjust the diameter and light intensity of the incident beam, and the two 632.8 nm wavelength narrow-band interference filters have the effect of eliminating the influence of stray light. Finally, the output of the differential photoelectric detector is displayed by the oscilloscope 28, and transferred to the computer to be processed.

One of the inputs of the photoelectric detector is put before the focusing point of the focusing lens, and the other one is put after the focusing point to form a differential signal. Adjustment of the mirror 2 and mirror 3 ensures the two light beams have an appropriate optical path difference, as well as the adjustment of the position of the 1:1 beam splitter should ensure the two lights, going into the differential photoelectric detector, have the same power. In absence of surface waves, the output of the differential photoelectric detector should be zero. When SAW signals are being detected by the He-Ne spot region, the reflected light will have a small trace offset for the change of the reflection angle, resulting in the change of relative beam intensity of the two beams incident to the differential photoelectric detector, thus detecting the surface acoustic wave signals.

The integrated laser-induced PZT/DC SAW system for Young's modulus measurement of thin films not only shows the advantages of piezoelectric LSAW detection technique, such as higher intensity of the signal amplitude and higher tolerance to optical disturbances, but also inherits the advantages of the differential confocal LSAW detection technique, such as rapid, accurate, higher measurement bandwidth, and higher signal-to-noise ratio, making it suitable for a wider range of applications, sample materials and measurement environments. This new integrated systems is more robust and promising for being used in production environments, improving the performance of traditional techniques.

### 3.2. Testing Scenarios and Operations Strategies

Users can freely select a detection mode in the integrated laser-induced PZT/DC SAW system according to the proper testing samples and scenarios, or even choose both techniques sequentially or at the same time to conduct cross-validation experiments, In the case of working synchronically, the SAWs detected by the two techniques can be displayed on the same oscilloscope, that's why users can compare the two results directly at the same time, however the results cannot be input and calculated by the computer automatically so far. The goal of the research team in the near future is to develop a DSP subsystem to considerably reduce the testing time. Table 2 lists the main application guidelines for the integrated system.

It can be seen from Table 2 that for different samples and measurement scenarios, the two detection paths have somehow different application areas. The new integrated laser-induced PZT/DC SAW system can harness the advantages of both techniques, greatly expanding the measurement range of the testing samples and scenarios. What's more, the integrated system can achieve cross-validation comparison measurements of Young's modulus without the need of conducting cross-validation with the nanoindentation technique. In addition, LSAW technique is a promising approach capable of measuring other mechanical and physical properties of thin films.

## 4. Conclusions

According to the main features of both LSAW detection approaches, namely, the piezoelectric and the differential confocal techniques, an integrated laser-induced PZT/DC SAW system was designed, constructed, and tested. The two detection channels of the integrated system share the same laser SAW excitation subsystem, and the integrated system is capable of performing accurate and sensitive measurements in a separated or simultaneous fashion. Depending on the different samples and testing scenarios, the integrated system can enhance and expand the range of Young's modulus measurement, giving also the capability to conduct efficient cross-validation measurements.

## Figures and Tables

**Table 1.** Characteristics of piezoelectric and differential confocal LSAW detection techniques.

**Characteristics**	**Piezoelectric LSAW detection**	**Differential confocal LSAW detection**
Signal detection method	Contact	Non-contact
Complexity of system	Low	Medium
Construction and calibration	Medium	Medium+
Capability to work in ultra-clean environments	Medium	High
Disturbance and interference tolerance	Medium [10,11]	Low [10,11] (sensitive to light, noise, vibration)
Type of sample materials	Layered, hard, semi-soft and porous solid materials	All layered, hard and soft/porous solid materials, except with high reflection and transparency
Potential to expand the type of testing sample material	Suitable for solid sample materials	It may be used for liquid and gaseous samples
Sample thickness	>0.5 mm [21]	Several nm to mm [7,10–12]
Thin film thickness	Several nm to mm [7,21]	Several nm to mm [7,10–12]
Surface roughness	R_a_ < 2 μm [21]	
Measurement bandwidth	∼120 MHz [9], ∼300 MHz [15,16]	∼300 MHz [10–12]
Relative error of Young's modulus measurement	1% [1]–30% [7]	∼1% [10]
Relative error of thickness measurement	1% [1]–10% [2]	1%–10% [20]
Relative error of porous density measurement	0.7% [7,12]	Not yet applied to the measurement of this variable
Signal-to-noise ratio	High [21]	High [11]
Repeatability	Medium [21]	High [12]
Probe Lifetime	Medium (requires often replacement due to PVDF foil aging) [21]	High (due to robust components and non-contact beam probe) [20]
Maintenance and Repair	The system is simple, easy to maintain, inspect and repair [20,21]	The system is more complex to maintain, inspect and repair [20]
Cost	Medium [20,21]	Medium-High [10]

**Table 2.** Application guidelines of the integrated laser-induced PZT/DC SAW system.

**Measurement Scenarios**	**Piezoelectric LSAW Detection Path**	**Differential Confocal LSAW det. Path**
Rough samples	Applicable	Applicable
Complex and irregular samples	NA, piezoelectric detector is big, difficult to place it when detection area is small	Applicable, the probe beam is a small spot, and its probe position is easy to locate
Samples with low reflection coefficient	Applicable	NA. The reflected probe light is too weak to detect SAW signals
Samples with high transparency	Applicable	NA. SAW detection is vulnerable to the influence of substrate properties
Sample materials are relatively soft such as biological surfaces	NA. Piezoelectric wedge influences the sample shape, introducing errors	Applicable
Samples with high porous density	NA. Piezoelectric wedge influences the measurement results	Applicable
Environment with different kind of disturbances	Applicable	NA. The optical system is susceptible and prone to interference, introducing error
Ultra-clean environment	Applicable, however the wedge contact technology may introduce pollution	Applicable
Static sample testing of IC's, MEMS manufacturing	Applicable	Applicable
Dynamic sample testing of IC's, MEMS manufacturing	NA	Applicable
Field test feasibility (*in situ*)	NA. The wedge probe and PVDF transducer is prone to vibrations	Applicable

NA—Not Applicable.
